# Temporal lobe epilepsy is associated with neuroinflammation, extracellular matrix remodeling, and synaptic protein alterations

**DOI:** 10.3389/fnmol.2026.1728666

**Published:** 2026-02-20

**Authors:** Sophia Auer, Lucas Hoffmann, Martin Schicht, Ingmar Blümcke, Friedrich Paulsen

**Affiliations:** 1 Institute of Functional and Clinical Anatomy, Friedrich-Alexander-University Erlangen-Nürnberg, Erlangen, Germany; 2 Department of Neuropathology, University Hospital Erlangen and FAU Erlangen-Nürnberg (European Reference Network (ERN) EpiCARE), Erlangen, Germany

**Keywords:** complement system, ECM remodeling, extracellular matrix, hippocampus, inflammation, proteomics, synapse, temporal lobe epilepsy

## Abstract

**Introduction:**

Temporal lobe epilepsy is the most prevalent form of drug-resistant focal epilepsy and is frequently associated with neuronal cell loss and astrogliosis in the hippocampus, i.e. hippocampal sclerosis (HS).

**Methods:**

In this study, we performed mass spectrometry-based proteomic profiling of microdissected hippocampal, neocortical, and white matter tissue obtained from TLE patients and respective control samples.

**Results:**

In hippocampal TLE tissue, we observed significant upregulation of proteins involved in complement system activation, extracellular matrix (ECM) organization, and astrocyte reactivity, indicative of active inflammatory remodeling within the sclerotic hippocampus. Conversely, synaptic proteins, including glutamate and gamma-aminobutyric acid (GABA) receptors, along with other regulators of synaptic structure and function, were markedly downregulated. Interestingly, in neocortical and white matter regions from the same TLE patients, immune- and ECM-related proteins were downregulated or unchanged, whereas synaptic proteins were preserved or upregulated.

**Discussion:**

These region-specific molecular signatures suggest that inflammatory-driven ECM remodeling is spatially restricted to the epileptogenic hippocampus, where it may contribute to synaptic destabilization and network dysfunction. Together, our findings support the hypothesis that inflammatory ECM remodeling in the hippocampus plays a central role in epileptogenesis in TLE. In contrast, the neocortical and white matter regions may undergo compensatory adaptions. The convergence of immune and ECM-related alterations on synaptic structures highlights a potential pathophysiological axis in epilepsy and points to novel molecular targets for therapeutic intervention.

## 1 Introduction

Focal epilepsies account for over 60% of all epilepsy cases globally, with temporal lobe epilepsy (TLE) being the most prevalent form in adults (Téllez-Zenteno and Hernández-Ronquillo, 2012). Within TLE, the mesial TLE (mTLE) accounts for approximately 80% of all TLE cases (Tatum, 2012). Notably, about one-third of the patients with mTLE develop drug-resistant epilepsy, which significantly limits treatment options. Epilepsy surgery with a resection of the epileptogenic zone represents the main therapeutic option for these patients. The most frequent feature of patients with mTLE is hippocampal sclerosis (HS), characterized by severe atrophy of the affected hippocampus due to neuronal cell loss, particularly of pyramidal cells in the cornu ammonis (CA) regions 1 and 4 of the hippocampal formation (Blümcke et al., 2002), as well as astrogliosis, which forms the basis of hippocampal scarring (Blümcke et al., 2013). Despite these pathological features, the molecular mechanisms underlying the pathogenesis of TLE are not fully understood, particularly those involving the innate immune response and ECM remodeling. Emerging evidence highlights the critical involvement of neuroinflammatory pathways, notably the complement system, in various central nervous system (CNS) disorders, including epilepsy (Gasque et al., 2000; Aronica et al., 2007; Schartz et al., 2018; Tenner et al., 2018; Lee et al., 2019; Gruber et al., 2022; Balduit et al., 2025). In both human TLE and animal models, the classical complement cascade is activated following status epilepticus (SE), with pronounced activation in brain regions exhibiting neuronal degeneration (Aronica et al., 2007). Sustained C1q-C3 signaling has been documented in rodent models of chronic epilepsy, further supporting the role for complement-mediated neuroinflammation in epilepsy (Schartz et al., 2018). Inflammatory processes and reactive astrocytosis have been implicated in epileptogenesis (van Vliet et al., 2018; Meng and Yao, 2020; Ravizza et al., 2024). A recent pathological classification introduced the TLE syndrome termed “innate inflammatory gliosis only” as a distinct subtype of TLE, characterized by prominent astrogliosis without neuronal cell loss (Grote et al., 2023). Notably, reactive astrocytes and inflammatory mediators are known to modulate ECM composition, which in turn may impair synaptic integrity and plasticity, thereby contributing to seizure generation and progression (Woo and Sontheimer, 2023). The ECM is a dynamic network that plays a crucial role in maintaining the structure and function of the central nervous system (CNS). Beyond providing structural support for neural cells, the ECM regulates physiological development and functions of the brain, such as synaptic plasticity, neuronal signaling, and neural development (Soles et al., 2023). These functions position the ECM as a central component of the tetrapartite synapse, facilitating proper synaptic transmission (Dityatev and Schachner, 2006; Dankovich and Rizzoli, 2022). It is reasonable to admit that remodeling processes of the ECM may disrupt the complex tetrapartite synaptic system, potentially leading to pathological states. Specifically, alterations of the ECM probably destabilize the synaptic system, resulting in an imbalance in excitation and inhibition, which may cause epilepsy (Dityatev, 2010; Chaunsali et al., 2021; Auer et al., 2025). Mass spectrometry-based proteomics has increasingly been used to characterize molecular alterations in neurological disorders (Sethi and Zaia, 2017; do Canto et al., 2021; Pires et al., 2021; Pokhilko et al., 2021; Chmelova et al., 2023; Downs et al., 2023; Leitner et al., 2024). In TLE, studies have applied quantitative proteomic techniques to analyze hippocampal tissue, identifying dysregulated proteins involved in eg., synaptic vesicle transport and synaptic functions, extracellular matrix organization, mitochondrial metabolic dynamics and function, immune response, neuroinflammation, and cell signaling (Zhang et al., 2020; Srivastava et al., 2024). Systematic reviews have summarized overlapping proteomic alterations, highlighting recurrent processes such as vesicle fusion, oxidative stress, cytoskeletal remodeling, and neurotransmitter regulation (Timechko et al., 2023). Despite these advances, most studies have focused on single regions, mainly the hippocampus, examined limited number of proteins, or lacked comparative analyses across multiple temporal lobe regions. To address these gaps, we extend previous work by performing an in-depth, region-resolved mass spectrometry-based proteomic analysis of microdissected hippocampal, neocortical, and white matter tissue from patients with TLE and non-epileptic controls. By profiling approximately 6000 proteins per sample and integrating pathway enrichment analyses, our study provides a comprehensive view of region-specific molecular signatures. Our findings reveal pronounced potentially inflammatory-driven ECM remodeling that may influence synaptic function in the sclerotic hippocampus (HC), in contrast to distinct patterns in neocortical (NCx) and white matter (WM) temporal regions. These results support the hypothesis that innate immune activation and ECM remodeling processes are spatially confined to the epileptogenic HC, where they may drive synaptic network dysfunction. Elucidating how neuroinflammation, ECM reorganization, and synaptic remodeling converge in a region-specific manner may help to uncover molecular mechanisms involved in epileptogenesis and identify potential targets for therapeutic intervention in epilepsy.

## 2 Material and methods

### 2.1 Ethics statement

The ethical review boards of the University of Erlangen approved the study under agreement number 22-209-BP.

### 2.2 Human brain tissue

We analyzed microdissected hippocampal (*n* = 4), neocortical (*n* = 4), and white matter (*n* = 4) tissue obtained from patients with TLE diagnosed with hippocampal sclerosis type 1 (HS1) undergoing epilepsy surgery. Corresponding control samples were included for each region (HC: *n* = 4; NCx: *n* = 3; WM: *n* = 4). Control tissues were derived from post-mortem cases and underwent routine neuropathological examination at the Department of Neuropathology, which revealed no specific neurological or neuropathological disease. One neocortical control sample was excluded following outlier analysis (data not shown). Detailed information on all samples, including brain region, diagnosis, age, and sex is provided in Tables 1, 2. All tissue samples, both from TLE patients and controls, were fresh-frozen immediately after extraction and stored at −80 °C as part of the European Epilepsy Brain Bank (Blumcke et al., 2017). In accordance with hospital and legal procedures, post-mortem intervals were at least 24 h. After death, bodies were stored at 4 °C until autopsy was legally confirmed.

**Table 1:** Control case information.

**Ctrl ID**	**Age**	**Sex**	**Cause of death**	**Brain region**
1	46	M	Liver cirrhosis	Hippocampus
1	46	M	Liver cirrhosis	Neocortex temporal lobe
1	46	M	Liver cirrhosis	White matter temporal lobe
2	47	F	Pulmonary embolism	Hippocampus
2	47	F	Pulmonary embolism	Neocortex temporal lobe
2	47	F	Pulmonary embolism	White matter temporal lobe
3	54	M	Volume deficiency due to blood loss	Hippocampus
3	54	M	Volume deficiency due to blood loss	Neocortex temporal lobe
3	54	M	Volume deficiency due to blood loss	White matter temporal lobe
4	51	M	Renal failure	Hippocampus
4	51	M	Renal failure	White matter temporal lobe

**Table 2:** TLE case information.

**TLE ID**	**Age at surgery**	**Sex**	**Neuropathology findings**	**Brain region**
1	39	F	TLE with Hippocampal sclerosis type 1	Hippocampus
1	39	F	TLE with Hippocampal sclerosis type 1	Neocortex temporal lobe
1	39	F	TLE with Hippocampal sclerosis type 1	White matter temporal lobe
2	33	M	TLE with Hippocampal sclerosis type 1	Hippocampus
2	33	M	TLE with Hippocampal sclerosis type 1	Neocortex temporal lobe
2	33	M	TLE with Hippocampal sclerosis type 1	White matter temporal lobe
3	17	M	TLE with Hippocampal sclerosis type 1	Hippocampus
3	17	M	TLE with Hippocampal sclerosis type 1	Neocortex temporal lobe
3	17	M	TLE with Hippocampal sclerosis type 1	White matter temporal lobe
4	20	F	TLE with Hippocampal sclerosis type 1	Hippocampus
4	20	F	TLE with Hippocampal sclerosis type 1	Neocortex temporal lobe
4	20	F	TLE with Hippocampal sclerosis type 1	White matter temporal lobe

### 2.3 Sample preparation

The mass spectrometry analysis was performed at the DTU Proteomics Core (Technical University of Denmark). 100 μl of lysis buffer (6M Guanidinium Hydrochloride, 10 mM TCEP, 40 mM CAA, 50 mM HEPES pH 8.5) was added to each sample along with a 3 mm tungsten carbide bead (Qiagen, Hilden, Germany). Samples were treated twice in the TissueLyser II homogenizer (Qiagen, Hilden, Germany) going from 3-30 Hz in 60 s. Beads were removed and samples were transferred to clean Eppendorf LoBind tubes (Eppendorf, Hamburg, Germany). Samples were boiled at 95 °C for 5 min, followed by sonication on high for 5 times 60 s on and 30 s off in a Bioruptor Pico sonication water bath (Diagenode, Liege, Belgium) at 4 °C. Samples were centrifuged at 18,000 RCF for 10 min, and supernatants were transferred to clean Eppendorf Protein LoBind tubes (Eppendorf, Hamburg, Germany). Protein concentrations were measured by BCA Rapid Gold (Thermo Scientific™, Waltham, USA) and 20 μg protein was taken forward for digestion. Sample volume was normalized between samples with additional lysis buffer and diluted 3x with digestion buffer (10% Acetonitrile in 50 mM HEPES pH 8.5) and 400 ng of LysC (MS-Grade Wako, Osaka, Japan) was added. Samples were incubated for 3 h at 37 °C while shaking at 750 RPM. After LysC digestion, samples were diluted to a final 10x in digestion buffer and digested with 200 ng Trypsin (MS-Grade, Osaka, Japan; Sigma Aldrich, St. Louis, USA) for 19 h at 37 °C while shaking at 750 RPM. Digestion was stopped by adding 2% trifluoroacetic acid (TFA) to a final concentration of 1%. The resulting peptides were desalted on a SOLAμ™ SPE plate (HRP, Thermo Scientific™, St. Louis, USA). Between each application, the solvent was spun through by centrifugation at 350 RCF. For each sample, the filters were activated with 200 μl of 100% methanol, then 200 μl of 80% acetonitrile, and 0.1% formic acid. The filters were equilibrated twice with 200 μl of 1% TFA, 3% acetonitrile, after which the samples were loaded. After washing the tips twice with 200 μl of 0.1% formic acid, the peptides were eluted into clean Eppendorf Protein LoBind tubes using 40% acetonitrile, 0.1% formic acid. The eluted peptides were concentrated in an Eppendorf Speedvac and reconstituted in 50 mM HEPES pH 8.5 for TMT labeling with 16plex tags (Thermo Scientific™, St. Louis, USA). A reference sample was prepared by mixing equal amounts of peptides from each sample and labeling that separately. Labeling was done according to manufacturer's instructions, and subsequently, labeled peptides were mixed, spiking in reference sample, resulting in two TMT pools. TFA was added to acidify and bring the acetonitrile concentration down to < 5%. Prior to mass spectrometry analysis, the peptides were desalted following the same procedure as describe previously and fractionated using an offline Thermo Scientific™ UltiMate™ 3,000 liquid chromatography system using high pH fractionation (5 mM Ammonium Bicarbonate, pH 10) at 5 μL/min flow rate (Thermo Scientific™, St. Louis, USA). Fifteen μg of each sample pool were separated over a 120-min gradient (5% to 35% acetonitrile), while collecting fractions every 130 s. The resulting 60 fractions were pooled into 30 final fractions, acidified to pH < 2 with 1% TFA and loaded onto EvoSep stage tips according to the manufacturer's protocol.

### 2.4 Mass spectrometry analysis

For each fraction, peptides were analyzed using the pre-set ‘30 samples per day' method on the EvoSep One instrument. Peptides were eluted over a 44-min gradient and analyzed with an Orbitrap Eclipse™ Tribrid™ instrument (Thermo Fisher Scientific™, St. Louis, USA) with FAIMS Pro™ Interface (ThermoFisher Scientific™, St. Louis, USA) switched between CVs of −50 V and −70 V with cycle times of 1.5 s. Full MS spectra were collected at a resolution of 1,20,000 with normalized AGC target set to “standard” or maximum injection time of 50 ms and a scan range of 375-1,500 m/z. Full MS spectra were collected at a resolution of 1,20,000, with normalized AGC target set to “standard” or maximum injection time of 50 ms and a scan range of 375–1,500 m/z. MS1 precursors with an intensity of >5 × 103 and charge state of 2-7 were selected for MS2 analysis. Dynamic exclusion was set to 60 s, the exclusion list was shared between CV values (Coefficient of Variation Values) and Advanced Peak Determination was set to “off”. The precursor fit threshold was set to 70% with a fit window of 0.7 m/z for MS2. Precursors selected for MS2 were isolated in the quadrupole with a 0.7 m/z window. Ions were collected for a maximum injection time of 50 ms and normalized AGC (Automatic Gain Control) target set to “standard”. Fragmentation was performed with a CID (Collision-induced dissociation) normalized collision energy of 35% and MS2 spectra were acquired in the IT at scan rate rapid. Precursors were subsequently filtered with an isobaric tag loss exclusion of TMT (Tandem Mass Tag) and precursor mass exclusion set to 18 m/z low and 5 m/z high. Precursors were isolated for an MS3 scan using the quadrupole with a 2 m/z window, and ions were collected for a maximum injection time of 86 ms and normalized AGC target of 200%. Turbo TMT was deactivated, and the number of dependent scans set to 5. Isolated precursors were fragmented again with 63% normalized HCD (Higher-energy Collision Dissociation) collision energy, and MS3 spectra were acquired in the orbitrap at 50,000 resolution with a scan range of 100-500 m/z. MS performance was verified for consistency by running a complex cell lysate quality control standard.

### 2.5 Data analysis

Biogenity (Contract Research Biotechnology Company, Aalborg, Denmark) contributed by performing the data analysis, including figures, tables, and reports. The raw files were analyzed using Proteome Discoverer 2.4 (Thermo Fisher Scientific™, St. Louis, USA). TMT reporter ion quantitation was enabled in the processing and consensus steps, and spectra were matched against the Homo sapiens database obtained from UniProt (Universal Protein Resource). Dynamic modifications were set as oxidation (M), and acetyl on protein N-termini. Cysteine carbamidomethyl (C) and TMT 16-plex (peptide N-termini and K) were set as static modifications. All results were filtered to a 1% FDR (False Discovery Rate) using the Benjamini-Hochberg procedure. Protein quantitation was done using the built-in Minora Feature Detector. Surgical and post-mortem control samples were processed using the same standardized proteomics workflow, including identical tissue lysis, digestion, TMT labeling, fractionation, and LC-MS/MS acquisition protocols. Samples were randomized across TMT channels and batches where possible, and pooled reference channels were included to enable cross-batch normalization. Quality control measures included assessment of labeling efficiency, peptide yield, reporter ion intensity distributions, and multivariate analyses to detect potential outliers or batch-driven effects. TMT reporter ion intensities were log_2_-transformed prior to downstream analysis. Sample-wise normalization was applied using global median (total signal) normalization across all TMT channels to correct for differences in protein loading and labeling efficiency. Missing values were not imputed prior to differential expression analysis. Proteins were retained only if they were quantified in the majority of samples per experimental group, in order to avoid introducing an artificial signal. The data was filtered so only proteins with 2 unique peptides and at least 60% valid values across all the samples, or at least 75% within an experimental group were used in the downstream data analysis. Statistical significance was assessed by either limma (Ritchie et al., 2015) or Wilcoxon signed-rank test depending on whether the proteins demonstrated a normal distribution or not. Filtration and statistical analysis were performed in the R programming language. Differential expression analysis was conducted using the limma package.

### 2.6 Enrichment analysis

For all datasets, gene set enrichment analysis (GSEA) was applied using ranked protein lists to detect coordinated pathway-level alterations in the proteomic dataset using the clusterProfiler R package (Korotkevich et al., 2016). Enrichment analysis was performed for Reactome pathway annotations for hippocampal, white matter and neocortical TLE tissue (Milacic et al., 2024). Pathway enrichment p-values in the GSEA Reactome analyses were adjusted for multiple testing. The corresponding enrichment tables for the most enriched pathways in hippocampal TLE tissue are provided in the Supplementary Tables 2, 3, and tables for neocortical and white matter regions can be made available upon request. For visualization of Reactome pathways enrichment results, waterfall plots were generated in R programming language using the ggplot2 and associated R packages. (Korotkevich et al., 2016).

## 3 Results

### 3.1 Proteomic analysis of TLE vs. control tissue

**Figure 1:**
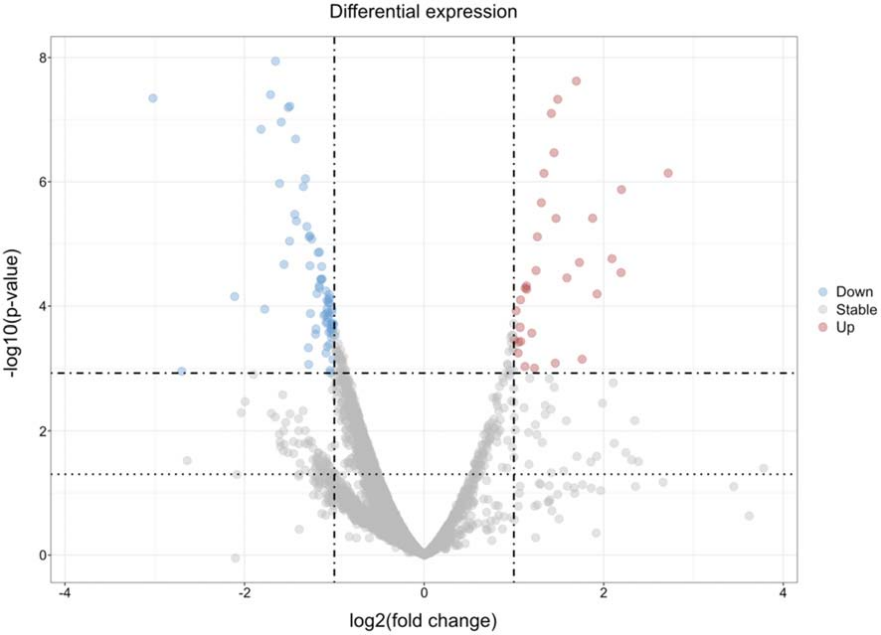
Volcano plot illustrating significantly differentially expressed proteins in hippocampal tissue in TLE. Mass-spectrometry based proteomic analysis of hippocampal tissue of TLE patients and respective control samples were analyzed statistically. The volcano plot illustrates significantly downregulated proteins and significantly upregulated proteins. X-axis shows the log_2_ fold change and y-axis denotates the p-value -log10. Red dots represent significantly upregulated proteins, whereas blue dots mark significantly downregulated proteins. The horizontal dashed lines depict the p-value threshold of 0.05 (lower line) and the adjusted p-value threshold of 0.05 (upper line). The vertical dashed line marks the threshold for an absolute log_2_ fold change ≥1. An interactive version is available on request at https://doi.org/10.5281/zenodo.18220542.

We performed mass-spectrometry-based analysis of hippocampal, temporal neocortical, and white matter samples from four TLE patients compared to location-matched controls to discover proteomic alterations under these chronic pathophysiological disease conditions. Histological evaluation confirmed TLE pathology with hippocampal sclerosis type 1 (HS type I, international consensus classification of hippocampal sclerosis in temporal lobe epilepsy, ILAE) across all pathologic samples [Table 2, (Blümcke et al., 2013)]. Control cases had no specific neuropathology findings (Table 1). Approximately 5,800 proteins were identified within each of the comparative sample groups (HC_Ctrl vs. HC_TLE; WM_Ctrl vs. WM_TLE; NCx_Ctrl vs. NCx_TLE, Supplementary Figure 1). This indicates a comparable quality of the tested samples. For exploratory analysis, proteins with a nominal *p* ≤ 0.05 and an absolute log2 fold change ≥±0.5 were considered differentially expressed. A more stringent log2 fold change cut-off of ≥ ± 0.9 was applied for proteins with an adjusted *p*-value of ≤ 0.05. In the comparison group of the hippocampal tissue, 1193 proteins showed significantly different expression with a *p*-value below or equal to 0.05 and a |log2 fold change| ≥0.5. One hundred and forty nine proteins were altered showing an adjusted *p*-value ≤ 0.05 after Benjamini-Hochberg procedure (Figure 1; Supplementary Table 1, row 1). In the comparison group of the white matter tissue, 1134 proteins showing altered expression levels with a *p*-value below or equal to 0.05 and a |log2 fold change| ≥ 0.5. Eighty proteins were significantly altered following adjusting for multiple comparisons with an adjusted *p*-value ≤ 0.05 and |log2 fold change| ≥ 0.9 (Supplementary Table 1, row 2; Supplementary Figure 2). When comparing the expression data of neocortical tissue from TLE patients and the respective control tissue, 109 proteins were altered with a *p*-value below or equal to 0.05 with |log2 fold change| ≥ 0.5. Thirteen proteins were still significantly altered after adjusting for multiple comparisons with an adjusted *p*-value ≤ 0.05 and absolute |log2 fold change| ≥ 0.9 (Supplementary Table 1, row 3; Supplementary Figure 3).

### 3.2 Differential protein expression in hippocampal TLE tissue compared to controls

**Figure 2:**
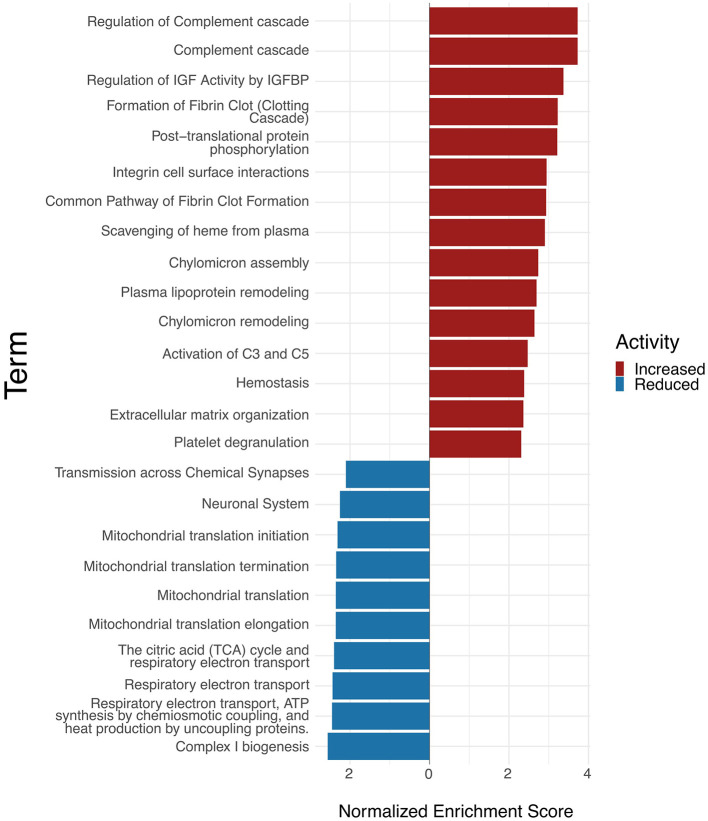
Gene set enrichment analysis (GSEA) of Reactome pathways in hippocampal TLE tissue. The waterfall plot depicts the enrichment of Reactome pathways within TLE conditions. The TOP25 altered terms are ranked by their normalized enrichment scores. The color of the bar denotes whether they are upregulated (red) or downregulated (blue). Performed using the clusterProfiler and ggplot2 R packages (Korotkevich et al., 2016; Milacic et al., 2024). Activity enrichment data is available on request. Abbreviations: IGF, Insulin-like Growth Factor; IGFBPs, IGF Binding Proteins.

Tables 3, 4 display up- and downregulated proteins in the hippocampal TLE tissue. Within the significantly upregulated proteins, several proteins of the complement system or immune system processes are shown to be altered in TLE (e.g., PPBP, C8B, C4B, C6, HRG, PGLYRP2, GPRC5B, ANXA1). Moreover, proteins associated with the ECM were upregulated in the epileptic hippocampal tissue, such as ECM receptor integrins (ITGA2B, ITGB3), the ECM receptor for hyaluronan CD44, as well as the ECM proteins THBS1 and TNC. Proteins expressed by astrocytes, including GFAP, CD44, and AQP4, were found to be upregulated in TLE, suggesting astrocyte activation in the epileptic hippocampus. Notably, several proteins involved in synaptic signaling and neurotransmission were downregulated in hippocampal tissue from TLE patients compared to controls. This included the expression of both glutamatergic and GABAergic receptor subunits, such as GABRA1, GRIN1, GRIN2B, GRIA1, GRIA2, GABBR2, GRM5, suggesting impaired excitatory and inhibitory neurotransmission. Additionally, proteins associated with synaptic vesicle transport, including SV2B, STXBP5L, STXBP5, SYP, SYNGAP1, SYNPR were also significantly downregulated. Key scaffolding and post synaptic density proteins such as DLG4, DLGAP1, DLGAP3, SHANK3, HOMER2, LRRTM1, SHISA6, and SHISA7 showed reduced abundance, indicating potential structural and functional disruption of synaptic structure. Furthermore, proteins involved in ion transport and calcium signaling displayed a decreased expression in TLE HC, including several potassium channel subunits (KCND2, KCND3, KCNAB1, KCNIP4). The glutamate transporter SLC17A7 (VGLUT1) was also significantly downregulated in TLE conditions in the HC. In parallel, several proteins related to mitochondrial and metabolic function (eg., NDUFS7, NDUFA3, NDUFA4) exhibited reduced expression, pointing to impaired mitochondrial function and potential oxidative stress in the epileptic HC. To annotate the most affected pathways among the differentially expressed proteins in HC TLE tissue, gene set enrichment analyses were performed (Supplementary Tables 2, 3) using Reactome pathway analysis (Korotkevich et al., 2016; Milacic et al., 2024). Among the significantly altered proteins in the epileptic HC of TLE patients, the most enriched pathways were associated with complement cascade activation and inflammatory responses, highlighting a prominent involvement of innate immune processes. In addition, the upregulation of proteins linked to extracellular matrix (ECM) organization suggests ongoing structural remodeling, potentially driven by neuroinflammatory mechanisms (Figure 2). Conversely, the most prominently downregulated pathways included those involved in chemical synaptic transmission and neuronal system functions, indicating potential impairments in synaptic activity. Pathways related to mitochondrial function were also downregulated, suggesting compromised energy metabolism and mitochondrial activity (Figure 2). However, this downregulation of synaptic and mitochondrial pathways may, at least in part, reflect neuronal cell loss characteristic of hippocampal sclerosis.

**Table 3:** Significantly upregulated proteins in hippocampal TLE tissue.

**Gene**	**UniprotID**	**Protein**	**Log_2_ fold change**	**limma p-value**
ITGA2B	P08514	Integrin alpha-IIb	2.7183826	7.2614E-07
PPBP	P02775	Platelet basic protein (PBP)	2.1976682	1.3362E-06
C8B	P07358	Complement component C8 beta chain	1.9260082	6.4052E-05
PGLYRP2	Q96PD5	N-acetylmuramoyl-L-alanine amidase	1.87686045	3.8572E-06
HRG	P04196	Histine-rich glycoprotein	1.7595509	0.00070311
ITGB3	P05106	Integrin beta-3	1.72936531	1.992E-05
GPRC5B	Q9NZH0	G-protein coupled receptor family C group 5 member B	1.69501241	2.3975E-08
CD44	P16070	CD44 antigen	1.4881553	4.717E-08
GFAP	P14136	Glial fibrillary acidic protein (GFAP)	1.41698924	7.9553E-08
AQP4	P55087	Aquaporin-4 (AQP-4)	1.33426181	7.3179E-07
C4B; C4B_2	P0C0L5	Complement C4-B (Basic complement C4)	1.30545567	2.1725E-06
C6	P13671	Complement component C6	1.19933854	0.00026717
ANXA1	P04083	Annexin A1	1.05174437	0.00037541
THBS1	P07996	Thrombospondin-1 (Glycoprotein G)	1.04736665	0.00055999
TNC	P24821	Tenascin C	0.96288584	0.00031551

The table lists the discussed or relevant (associated with synaptic system, mitochondrial processes, ECM, or immune system) proteins identified in the dataset, along with associated statistical measures. Primary gene name: Official gene name corresponding to the encoded protein according to UniProt database (Bateman et al., 2025). UniProt ID: Unique protein identifier from UniProt database. Protein name: Full name of the encoded protein according to UniProt annotation. Log_2_ fold change: Log_2_-transformed ratio of protein abundance between comparison groups (positive values indicate upregulation and negative values indicate downregulation). Limma p-value: Statistical significance of differential expression as calculated by limma package (Ritchie et al., 2015).

**Table 4:** Significantly downregulated proteins in hippocampal TLE tissue.

**Gene**	**UniprotID**	**Protein**	**Log_2_ fold change**	**limma p-value**
KCND2	Q9NZV8	Potassium voltage-gated channel subfamily D member 2 (Voltage-gated potassium channel subunit Kv4.2)	−2.110181	7.021E-05
NDUFAF4	Q9P032	NADH dehydrogenase [ubiquinone] 1 alpha subcomplex assembly factor 4	−1.3443366	1.1972E-06
GRIA1	P42261-6	Glutamate receptor 1 (GluR-1)	−1.3212101	8.9484E-07
NDUFAF3	Q9BU61	NADH dehydrogenase [ubiquinone] 1 alpha subcomplex assembly factor 3	−1.3049891	5.2614E-06
KCNAB1	Q14722	Voltage-gated potassium channel subunit beta-1	−1.265779	0.00013195
HOMER2	Q9NSB8	Homer protein homolog 2 (Homer-2) (Cupidin)	−1.2513606	8.3475E-06
SYNPR	Q8TBG9	Synaptoporin	−1.1809593	1.3786E-05
LRRTM1	Q86UE6	Leucine-rich repeat transmembrane neuronal protein 1	−1.167807	4.6814E-05
KCNIP4	Q6PIL6	Kv channel-interacting protein 4	−1.1511604	3.7094E-05
SLC17A7	Q9P2U7	Vesicular glutamate transporter 1	−1.1462432	3.6973E-05
GRIN1	Q05586	Glutamate receptor ionotropic, NMDA 1 (GluN1)	−1.1406	2.3155E-05
SHISA6	Q6ZSJ9	Protein shisa-6	−1.1157741	0.0001408
DLGAP3	O95886	Disks large-associated protein 3	−1.0835619	8.0725E-05
GABBR2	O75899	Gamma-aminobutyric acid type B receptor subunit 2 (GABA-B receptor 2)	−1.0755942	0.00018265
NDUFA3	O95167	NADH dehydrogenase [ubiquinone] 1 alpha subcomplex subunit 3	−1.0738047	0.00012609
SHISA7	A6NL88	Protein shisa-7	−1.073653	0.00015209
SYP	P08247	Synaptophysin (Major synaptic vesicle protein p38)	−1.0648625	8.6638E-05
GRIA2	P42262	Glutamate receptor 2 (GluR-2) (AMPA-selective glutamate receptor 2)	−1.0566118	6.5326E-05
STXBP5	Q5T5C0	Syntaxin-binding protein 5	−1.056457	0.00014117
SYNGAP1	Q96PV0	Ras/Rap GTPase-activating protein SynGAP (Neuronal RasGAP)	−1.0554713	7.3959E-05
DLG4	P78352	Disks large homolog 4 (Postsynaptic density protein 95) (PSD-95)	−1.0058289	0.00019541
SV2B	Q7L1I2	Synaptic vesicle glycoprotein 2B	−0.9435778	0.00058133
DLGAP1	O14490	Disks large-associated protein 1 (DAP-1)	−0.9275904	0.00075294
GRM5	P41594	Metabotropic glutamate receptor 5 (mGluR5)	−0.9273289	0.00068288
STXBP5L	Q9Y2K9	Syntaxin-binding protein 5-like	−0.9235621	0.00112422
SHANK3	Q9BYB0	SH3 and multiple ankyrin repeat domains protein 3	−0.9235588	0.00068885
NDUFS7	O75251	NADH dehydrogenase [ubiquinone] iron-sulfur protein 7	−0.9159593	0.00073938
GRIN2B	Q13224	Glutamate receptor ionotropic, NMDA 2B (GluN2B)	−0.9047638	0.00091014
GABRA1	P14867	Gamma-aminobutyric acid receptor subunit alpha-1	−0.8997057	0.00109861

The table lists discussed, or relevant (associated with synaptic system, mitochondrial processes, ECM, or immune system) proteins identified in the dataset, along with associated statistical measures. Primary gene name: Official gene name corresponding to the encoded protein according to UniProt database (Bateman et al., 2025). UniProt ID: Unique protein identifier from UniProt database. Protein name: Full name of the encoded protein according to UniProt annotation. Log_2_ fold change: Log_2_-transformed ratio of protein abundance between comparison groups (positive values indicate upregulation and negative values indicate downregulation). Limma p-value: Statistical significance of differential expression as calculated by limma package (Ritchie et al., 2015).

### 3.3 Differential protein expression in white matter TLE tissue compared to controls

**Figure 3:**
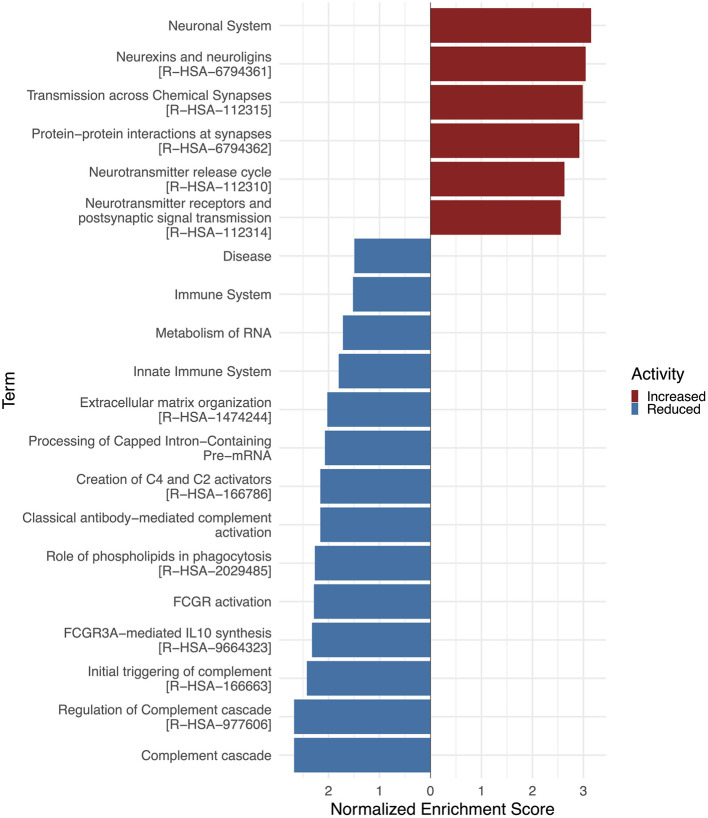
Gene set enrichment analysis (GSEA) of Reactome pathways in white matter TLE tissue. The waterfall plot depicts the enrichment of Reactome pathways within TLE conditions. The TOP20 altered terms are ranked by their normalized enrichment scores. The color of the bar denotes whether they are upregulated (red) or downregulated (blue). Performed using the clusterProfiler and ggplot2 R packages (Korotkevich et al., 2016; Milacic et al., 2024). Activity enrichment data is available on request. Abbreviations: FCGR, Fc gamma receptor.

In this study, the white matter TLE tissue displayed significantly altered proteins when comparing to control cases (Supplementary Figure 2). The most relevant and significantly altered proteins are listed in Tables 5, 6. Several upregulated proteins were directly involved in synaptic transmission and plasticity, including synaptic proteins (LRRTM2, CPLX3, SYT5, SYT6, CACNA1B, NPTX2, NPTXR) and glutamatergic receptors (GRIK3, GRM2). Additionally, synapse-associated signaling molecules were upregulated (GPRIN3, PLXNA5, SEMA4A, LINGO1) in the white matter of TLE patients. Our enrichment analysis using Reactome database revealed significant upregulation of proteins associated with the neuronal system, including transmission across chemical synapses, protein-protein interactions at synapses, and neurotransmitter signaling processes. In contrast, proteins annotated to the pathway ECM organization, including structural ECM components such as ACAN, VCAN, TNC, FBLN1, LAMA2, LAMB2, SPOCK, SPOCK1), and integrin-mediated ECM receptors (ITGB1, ITGA7), were downregulated. Moreover, complement and immune system associated proteins also displayed downregulated expression in white matter TLE tissue (eg., S100A8, S100A9, PRNP, C2, CFI, CFH, C3, C5, C6) (Figure 3). These findings suggested enhanced synaptic activity and potential synaptogenesis in the white matter of TLE patients, accompanied by ECM remodeling. Notably, these molecular alterations oppose the signatures observed in the hippocampal tissue, highlighting region-specific pathomechanisms in TLE.

**Table 5:** Significantly upregulated proteins in white matter TLE tissue.

**Gene**	**UniprotID**	**Protein**	**Log_2_ fold change**	**limma *p*-value**
DTNA	Q9Y4J8-10	Dystrobrevin alpha (DTN-A) (Alpha-dystrobrevin)	2.06063488	0.0002705
CPLX3	Q8WVH0	Complexin-3 (Complexin III) (CPX III)	1.93605507	0.01839235
LINGO1	Q96FE5	Leucine-rich repeat and immunoglobulin-like domain-containing nogo receptor-interacting protein 1	1.60997896	0.02348878
GPRIN3	Q6ZVF9	G protein-regulated inducer of neurite outgrowth 3 (GRIN3)	1.51520631	0.0244484
SEMA4A	Q9H3S1	Semaphorin-4A (Semaphorin-B) (Sema B)	1.3739658	0.00040583
NPTXR	O95502	Neuronal pentraxin receptor	1.35641044	0.01669707
GRIK3	Q13003	Glutamate receptor ionotropic. kainate 3 (GluK3)	1.29102414	0.04811656
NPTX2	P47972	Neuronal pentraxin-2 (NP2)	1.19924895	0.01337159
CACNA1B	Q00975	Voltage-dependent N-type calcium channel subunit alpha-1B	1.13524617	0.01555218
SYT5	O00445	Synaptotagmin-5 (Synaptotagmin V) (SytV)	0.95887317	0.00463861
PLXNA4	Q9HCM2	Plexin-A4	0.85887865	0.02725051
LRRTM2	O43300	Leucine-rich repeat transmembrane neuronal protein 2	0.72489571	0.0401921
SYT6	Q5T7P8	Synaptotagmin-6 (Synaptotagmin VI) (SytVI)	0.68888906	0.04437861
GRM2	Q14416	Metabotropic glutamate receptor 2 (mGluR2)	0.67641698	0.04984859

The table lists discussed, or relevant proteins (associated with synaptic system, ECM, or immune system) identified in the dataset, along with associated statistical measures. Primary gene name: Official gene name corresponding to the encoded protein according to UniProt database (Bateman et al., 2025). UniProt ID: Unique protein identifier from UniProt database. Protein name: Full name of the encoded protein according to UniProt annotation. Log_2_ fold change: Log_2_-transformed ratio of protein abundance between comparison groups (positive values indicate upregulation and negative values indicate downregulation). Limma p-value: Statistical significance of differential expression as calculated by limma package (Ritchie et al., 2015). Proteins with nominal significance (p ≤ 0.05) were also included (highlighted).

**Table 6:** Significantly downregulated proteins in white matter TLE tissue.

**Gene**	**UniprotID**	**Protein**	**Log_2_ fold change**	**limma p-value**
CTSG	P08311	Cathepsin G (CG)	−2.5231665	2.503E-07
S100A9	P0702	Protein S100-A9	−1.8201783	7.9131E-08
S100A8	P05109	Protein S100-A8	−1.5886123	2.7432E-06
C2	P06681	Complement C2 (EC 3.4.21.43) (C3/C5 convertase)	−1.5229096	0.00031092
PRNP	P04156	Major prion protein (PrP)	−1.387944	1.5695E-06
ITGA2	P17301	Integrin alpha-2	−1.2871631	0.00251664
HTRA1	Q92743	Serine protease HTRA1	−1.2321252	0.00026444
CFI	P05156	Complement factor I	−1.2302488	0.00030401
CFH	P08603	Complement factor H (H factor 1)	−1.1671839	0.04432039
FBLN1	P23142	Fibulin-1 (FIBL-1)	−1.112044	0.00029374
C3	P01024	Complement C3	−1.0519102	0.03470235
VCAN	P13611-3	Versican core protein	−1.0065955	0.00024766
TNC	P24821	Tenascin (TN) (Cytotactin)	−0.9614239	0.0005009
C5	P01031	Complement C5	−0.9321672	0.03171404
SPOCK3	Q9BQ16	Testican-3	−0.9289697	0.00446799
C6	P13671	Complement component C6	−0.8438197	0.01073331
ITGA7	Q13683	Integrin alpha-7	−0.8404531	0.00243328
ACAN	P16112	Aggrecan core protein	−0.8146514	0.00398191
ITGB1	P05556	Integrin beta-1 (Fibronectin receptor subunit beta)	−0.8014504	0.00077582
SPOCK1	Q08629	Testican-1 (Protein SPOCK)	−0.7936972	0.00125797
LAMA2	P24043	Laminin subunit alpha-2	−0.6861524	0.00274338
LAMB2	P55268	Laminin subunit beta-2	−0.6850167	0.00405122

The table lists discussed, or relevant proteins (associated with synaptic system, ECM, or immune system) proteins identified in the dataset, along with associated statistical measures. Primary gene name: Official gene name corresponding to the encoded protein according to UniProt database (Bateman et al., 2025). UniProt ID: Unique protein identifier from UniProt database. Protein name: Full name of the encoded protein according to UniProt annotation. Log_2_ fold change: Log_2_-transformed ratio of protein abundance between comparison groups (positive values indicate upregulation and negative values indicate downregulation). Limma p-value: Statistical significance of differential expression as calculated by limma package (Ritchie et al., 2015). Proteins with nominal significance (p ≤ 0.05) were also included (highlighted).

### 3.4 Differential protein expression in neocortical TLE tissue compared to controls

**Figure 4:**
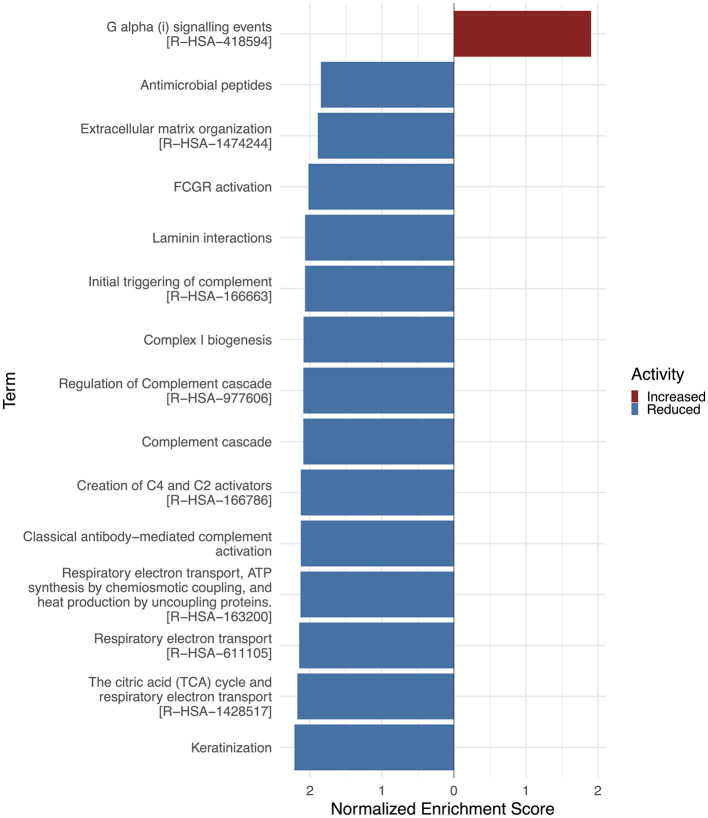
Gene set enrichment analysis (GSEA) of Reactome pathways in neocortical TLE tissue. The waterfall plot depicts the enrichment of Reactome pathways within TLE conditions. The TOP15 altered terms are ranked by their normalized enrichment scores. The color of the bar denotes whether they are upregulated (red) or downregulated (blue). Performed using the clusterProfiler and ggplot2 R packages (Korotkevich et al., 2016; Milacic et al., 2024). Activity enrichment data is available on request.

In the temporal neocortex (NCx) of TLE patients, only few proteins were significantly altered (Tables 7, 8, Supplementary Figure 3). Notably, we observed downregulation of pathways related to ECM organization (eg., MMP9, HAPLN4) and the immune system, including components of the complement cascade (such as S100A8, S100A9, C1QB), consistent with the patterns seen in the white matter tissue (Figure 4). A few synapse-associated proteins displayed nominal upregulation (e. g. NPTXR, SYT17, GRIK3, NPTX2, SYT6, DLG2). Overall, these limited protein changes provide only modest biological insight, suggesting that the NCx is less affected by epileptogenic processes and may exhibit compensatory responses.

**Table 7:** Significantly upregulated proteins in neocortical TLE tissue.

**Gene**	**UniprotID**	**Protein**	**Log_2_ fold change**	**limma *p*-value**
SSTR2	P30874	Somatostatin receptor type 2	3.19806355	1.5137E-14
FBLN2	P98095	Fibulin-2 (FIBL-2)	1.28275321	0.00045701
GRIK3	Q13003	Glutamate receptor ionotropic. kainate 3	0.94326662	0.00855291
NPTXR	O95502	Neuronal pentraxin receptor	0.93828035	0.0084982
FAM107A	O95990	Actin-associated protein FAM107A	0.89008964	0.01462238
SYT17	Q9BSW7	Synaptotagmin-17	0.87816741	0.0131029
GPRIN3	Q6ZVF9	G protein-regulated inducer of neurite outgrowth 3 (GRIN3)	0.84405854	0.01857629
NPTX2	P47972	Neuronal pentraxin-2 (NP2)	0.81933626	0.02138761
DLG2	Q15700-4	Disks large homolog 2	0.77989507	0.04211012
SYT6	Q5T7P8	Synaptotagmin-6	0.74938652	0.03998333
CSPG5	O95196	Chondroitin sulfate proteoglycan 5	0.73552322	0.03793015

The table lists discussed, or relevant proteins (associated with synaptic system, ECM, or immune system) proteins identified in the dataset, along with associated statistical measures. Primary gene name: Official gene name corresponding to the encoded protein according to UniProt database (Bateman et al., 2025). UniProt ID: Unique protein identifier from UniProt database. Protein name: Full name of the encoded protein according to UniProt annotation. Log_2_ fold change: Log_2_-transformed ratio of protein abundance between comparison groups (positive values indicate upregulation and negative values indicate downregulation). Limma p-value: Statistical significance of differential expression as calculated by limma package (Ritchie et al., 2015). Proteins with nominal significance (p ≤ 0.05) were also included (highlighted).

**Table 8:** Significantly downregulated proteins in neocortical TLE tissue.

**Gene**	**UniprotID**	**Protein**	**Log_2_ fold change**	**limma *p-*value**
S100A8	P05109	Protein S100-A8 (Calgranulin-A)	−2.7145045	1.9013E-14
S100A9	P06702	Protein S100-A9 (Calgranulin-B)	−2.5401516	7.7874E-13
MMP9	P14780	Matrix metalloproteinase-9 (MMP-9)	−1.4922929	8.1098E-05
HAPLN4	Q86UW8	Hyaluronan and proteoglycan link protein 4	−1.3585865	0.00016315
HTRA1	Q92743	Serine protease HTRA1	−0.7881468	0.02631057
C1QB	P02746	Complement C1q subcomponent subunit B	−0.7503325	0.03435565

The table lists discussed, or relevant proteins (associated with synaptic system, ECM, or immune system) proteins identified in the dataset, along with associated statistical measures. Primary gene name: Official gene name corresponding to the encoded protein according to UniProt database (Bateman et al., 2025). UniProt ID: Unique protein identifier from UniProt database. Protein name: Full name of the encoded protein according to UniProt annotation. Log_2_ fold change: Log_2_-transformed ratio of protein abundance between comparison groups (positive values indicate upregulation and negative values indicate downregulation). Limma p-value: Statistical significance of differential expression as calculated by limma package (Ritchie et al., 2015). Proteins with nominal significance (p ≤ 0.05) were also included (highlighted).

## 4 Discussion

In this study, we conducted a comprehensive analysis of region-specific proteomic alterations in TLE brain tissue to gain deeper insights into molecular mechanisms associated with the epileptogenic disease condition. The present findings supported the hypothesis that neuroinflammatory processes may contribute to pathologic ECM remodeling within the epileptogenic hippocampus, potentially leading to alterations in synaptic protein expression and disruption of functional neural networks, key features of the epileptic phenotype (Aronica et al., 2007, 2017; van Vliet et al., 2018; Gruber et al., 2022; Grote et al., 2023; Woo and Sontheimer, 2023; Ravizza et al., 2024). In contrast, the temporal neocortex and white matter exhibited proteomic signatures suggestive of compensatory or adaptive responses, highlighting regional heterogeneity in TLE pathology. Hippocampal sclerosis, most commonly ILAE type 1, is a hallmark of TLE and is characterized by neuronal cell loss predominantly in the CA1 and CA4 region of the HC and reactive astrogliosis (Blümcke et al., 2013). Our proteomic analysis supports these observations, showing elevated levels of glial fibrillary acid protein (GFAP), an established marker for astrocytic activation (Blümcke et al., 2013). In addition, other astrocyte-associated proteins such as S100A6 and the water channel aquaporin-4 (AQP4) were significantly upregulated, in line with previous studies linking their expression to astrogliosis and disrupted water homeostasis in TLE (Lee et al., 2004; Das et al., 2012; Jurewicz et al., 2013). Reactive astrocytes are known to both respond to and regulate complement system activation, which has been implicated in various CNS pathologies (Gasque et al., 2000; Aronica et al., 2007; Schartz et al., 2018; Tenner et al., 2018; Lee et al., 2019; Gruber et al., 2022; Grote et al., 2023). In our study, we observed significant upregulation of complement components C8B, C4B, and C6 (adjusted *p* < 0.05), with additional complement proteins showing nominal significance (*p* < 0.05), indicating robust innate immune activation in the epileptogenic hippocampus. These findings align with data from rodent models of TLE and human studies, as well as proteomic studies demonstrating persistent complement activation (Aronica et al., 2007; Timechko et al., 2023). Inflammatory processes, including complement activation, have been shown to drive ECM remodeling or reorganization in various pathological conditions (Sjöberg et al., 2005; Happonen et al., 2012; Biasella et al., 2023; Balduit et al., 2025). Our proteomic analysis revealed significant alterations in ECM-associated proteins, including upregulation of ECM receptor integrins. Alterations in integrin expression have been implicated in disrupted synaptic transmission, contributing to neurological disorders such as epilepsy, Alzheimer's disease, and schizophrenia (Wu and Reddy, 2012). Gene expression studies in epilepsy models also support the activation of ECM-integrin pathways under epileptic conditions within the HC (Han et al., 2019). Furthermore, we observed increased expression of the astrocytic hyaluronan receptor CD44, which anchors hyaluronan-based ECM, particularly perineuronal nets (PNNs), to astrocytic processes and thereby contributing to the formation of the tetrapartite synapse (Dankovich and Rizzoli, 2022; Auer et al., 2025). This is also in agreement with the recently described increase of PNNs in the epileptogenic human hippocampus (Lehner et al., 2024). Furthermore, dysregulated expression of CD44 has been implicated in various neurological disorders, including epilepsy and Alzheimer's disease (Akiyama et al., 1993; Borges et al., 2004; Bausch, 2006; Li et al., 2020; Kruk et al., 2023), and its upregulation in TLE may contribute to ECM restructuring. Notably, CD44 deficiency has been shown to reduce seizure severity in experimental models, underscoring its potential role in epileptogenesis (Kruk et al., 2023). Our findings also show significant upregulation of the ECM glycoprotein tenascin-C (TNC) in hippocampal TLE tissue. TNC expression has been linked to seizure activity and TGF-β-signaling pathway activation in rodent epilepsy models (Ferhat et al., 1996; Mercado-Gómez et al., 2014). Similarly, thrombospondin-1 (THBS1) was elevated in our data and has previously been implicated in the formation of epilepsy through its interaction with various pathways involved in processes such as synaptogenesis, oxidative stress, or calcium homeostasis (Christopherson et al., 2005; Risher and Eroglu, 2012; Cheng et al., 2024). Additionally, previous proteomic analysis revealed altered expression of ECM proteins such as THBS1 in the HC of TLE patients, further supporting the role of ECM reorganization leading to altered excitability in epilepsy (Srivastava et al., 2024). Collectively, these findings support the hypothesis that astrocytic activation, complement-mediated neuroinflammation, and ECM remodeling interact to create a pathologic environment in the sclerotic hippocampus, potentially driving synaptic dysfunction and contributing to the pathogenesis of TLE. However, this hypothesis suggests potential causal relationships, and in the absence of mechanistic data, these interpretations should be considered with caution. While these findings highlighted the upregulation of inflammatory and ECM-associated proteins in the epileptic HC, our analysis also revealed distinct downregulated proteins, primarily related to synaptic signaling and mitochondrial function, suggesting dysfunction or neuronal communication and energy metabolism in TLE. Notably, we observed significant downregulation of potassium channel subunits, along with their interacting protein KCNIP4. These channels have been previously implicated in regulating neuronal excitability and contributing to the pathogenesis of epilepsy (Simeone et al., 2013; Köhling and Wolfart, 2016; Liu et al., 2020; Zhang et al., 2023). In addition, several glutamate receptor subunits and GABA receptor components exhibited reduced expression, suggesting impaired excitatory and inhibitory neurotransmission. In our study, we observed significant downregulation of the glutamate transporter SLC17A7 (VGLUT1), consistent with previous studies reporting reduced VGLUT1 protein levels in hippocampal subfields with pronounced neuronal loss in HS patients (Van Der Hel et al., 2009). In a recent proteomic study, reduced VGLUT1 levels were correlated with impaired packaging of vesicular neurotransmitters in TLE with HS (Zhang et al., 2020). Moreover, numerous synapse-associated proteins, particularly those involved in postsynaptic density, and synaptic scaffolding, were significantly decreased in TLE hippocampi, pointing to synaptic dysfunction or loss. These changes may reflect or result from inflammatory-driven ECM remodeling and associated structural disruptions at the synaptic level. However, further studies are needed to evaluate synaptic integrity and function in the epileptic HC, as the observed changes may partially reflect neuronal loss associated with hippocampal sclerosis rather than direct molecular downregulation. Furthermore, our findings suggest mitochondrial dysfunction in the hippocampal tissue of TLE patients, as evidenced by the downregulation of proteins associated with energy metabolism. Pathway enrichment analysis of the significantly downregulated proteins revealed a strong enrichment for pathways related to aerobic respiration and mitochondrial processes, supporting the presence of mitochondrial impairments. These alterations may be secondary to neuroinflammatory mechanisms seen in hippocampal TLE tissue. Mitochondrial dysfunction and aberrant energy metabolism have previously been implicated in epilepsy (Rowley and Patel, 2013; Grove et al., 2020; Qin et al., 2024; Su et al., 2024a,b), and proteomic analyses have also reported dysregulation of mitochondrial proteins in epileptic brain tissue (Pires et al., 2021; Xiao et al., 2021; Srivastava et al., 2024). For example, proteomic profiling of the dentate gyrus from patients with HS identified downregulated components of oxidative phosphorylation, including subunits of mitochondrial complexes, which are directly involved in energy metabolism (Xiao et al., 2021). Our findings further support a link between neuroinflammation and mitochondrial impairment in epileptic brain and align with proteomic evidence for altered mitochondrial function in TLE. Nonetheless, further studies are warranted to elucidate the interplay between neuroinflammation and mitochondrial dysfunction in TLE, as this downregulation might be due to neuronal cell loss in the hippocampus. Compared to the epileptic hippocampal tissue, the temporal white matter and neocortex exhibited fewer significant proteomic alterations, limiting the interpretability and biological conclusions that can be drawn from these regions. Still, the NCx and WM exhibited in some cases opposing proteomic alterations to the HC. Pathway enrichment analysis indicated downregulation of the complement cascade (eg., C2, CFI, CFH, C3, C5, C6, C1QB) and immune response (S100A9, S100A8, PRNP) in both regions, indicating that neuroinflammatory processes are more strongly localized in the hippocampal seizure focus, while temporal regions are less affected. Furthermore, proteins annotated to ECM organization were downregulated within both regions in TLE (eg., CTSG, ACAN, VCAN, TNC, CTSG, Integrins, HAPLN4, and MMP9). The ECM protease MMP9 has been associated with epilepsy in various other studies. Elevated MMP9 levels were associated with neuronal death, aberrant synaptic plasticity, and neuroinflammation during epileptogenesis (Wilczynski et al., 2008; Heuser et al., 2010; Mizoguchi and Yamada, 2013; Acar et al., 2015). In contrast to these reports, our data did not reveal increased MMP9 expression in the HC, but rather a relative reduction in the NCx. This may reflect region-specific differences in MMP9 dependent processes, possibly indicating a compensatory in the neocortical region. In contrast to these downregulated processes, several synaptic proteins displayed upregulation in the white matter TLE tissue (e. g. GRIK3, SYT5, SYT6, GRM2), possibly reflecting compensatory synaptogenesis or network remodeling in response to hippocampal network dysfunction. Notably, the presence and role of synapses in the white matter of TLE patients have been explored in prior studies (Sóki et al., 2022; Yang et al., 2023), although their exact role remains unclear and warrants further investigation. In the NCx, nominal upregulation of synaptic proteins, such as NPTXR, SYT17, GRIK3, NPTX2, SYT6, and DLG2, was observed, but the low number of significant changes in this region limits interpretability. Overall, these findings suggest that while both NCx and WM exhibit only few molecular alterations in TLE, the HC shows the most pronounced changes, consistent with its central role as epileptogenic focus. Proteomic changes in NCx and WM may represent compensatory adaptions, emphasizing the region-specificity of molecular pathology in TLE.

### 4.1 Conclusion and outlook

In summary, our findings highlight the potential role of innate immune activation in the pathophysiology of TLE, as evidenced by the upregulation of complement components C8B, C4B, and C6. The data further suggest that neuroinflammatory processes may drive pathological remodeling of the ECM, including increased expression of integrins, CD44, thrombospondin-1 (THBS1), and tenascin C (TNC). These changes likely compromise the structural and functional integrity of hippocampal networks, as reflected by downregulation of key synaptic proteins such as synaptophysin (SYP), HOMER2, and neurotransmitter receptors, e. g. GABA and glutamate receptors. Proteomic profiling underscores the HC as central region involved in seizure generation, characterized by upregulation of astrocytic, complement, and ECM-related proteins alongside a reduction in synaptic and mitochondrial proteins. Collectively, these alterations point to a pathological cascade in which astrocytic activation and complement-mediated inflammation promote ECM remodeling, synaptic disruption, and impaired energy metabolism–interconnected mechanisms that may contribute to TLE pathology. Given the exploratory nature of this study, the findings should be interpreted with caution. Further experimental validation is essential to confirm the functional relevance of the identified proteomic changes and to evaluate their potential as biomarkers or therapeutic targets in TLE.

### 4.2 Limitations

The present study provides valuable insights into the proteomic alterations associated with TLE, with a particular emphasis on neuroinflammation, ECM, and synaptic changes, all of which are likely relevant for epilepsy pathogenesis. However, several limitations must be acknowledged. First, the relatively small sample size limits the statistical power and generalizability of the findings, as access to human tissue suitable for high-sensitivity mass spectrometry analysis is limited. Second, control tissues were obtained postmortem, whereas TLE tissues were surgically resected. While efforts were made to minimize batch effects, differences in tissue source, postmortem delay, and processing could introduce variability. Third, slight age differences between control and TLE samples may introduce variability, and obtaining perfectly age-matched, neuropathology-free controls is challenging. Furthermore, the high degree of individual variability, with each sample possessing a unique molecular signature, adds complexity to direct comparisons and may influence the observed proteomic changes. Fourth, the proteomic data are correlative, and causal relationships between inflammation, ECM remodeling, and synaptic and mitochondrial alterations cannot be inferred. Downregulation of synaptic and mitochondrial proteins in the hippocampus may partly reflect neuronal loss due to hippocampal sclerosis rather than direct molecular downregulation. Finally, no validation using additional techniques was performed to confirm the proteomic findings. A limitation of pathway enrichment analyses is their reliance on curated databases that annotate proteins based on their shared molecular functions and interaction networks rather than tissue specificity or disease context. As a result, pathway enrichment analyses may yield terms that appear unexpected in brain tissue. Importantly, presence of such pathways does not imply direct involvement of these processes in epilepsy but rather reflects overlapping molecular components that also participate in neuroinflammatory signaling, or cellular stress response within the central nervous system. Therefore, our discussion focuses on biologically relevant processes in CNS pathology and epileptogenesis, including neuroinflammation, ECM, synaptic dysfunction, and mitochondrial impairment. Despite these limitations, this study offers novel perspectives into inflammatory-driven ECM and synaptic remodeling processes in epilepsy. The application of ultra-sensitive mass spectrometry enabled an in-depth proteomic analysis of human tissue, and the examination of different brain regions provided valuable information on region-specific alterations in TLE. Future studies with larger cohorts and complementary validation techniques will be essential to confirm these findings and further elucidate our understanding of molecular mechanisms underlying ECM and synaptic changes in TLE.

## Data Availability

The mass spectrometry data generated in this study have been deposited to the ProteomeXchange Consortium via the PRIDE (Perez-Riverol et al., 2025) partner repository with the dataset identifier PXD068312.

## Supplementary material

Supplementary Figure 1 Bar chart total amount of identified proteins.

Supplementary Figure 2 Volcano plot white matter TLE tissue.

Supplementary Figure 3 Volcano plot neocortical TLE tissue.

Supplementary Table 1 Statistical Analysis of proteomic differences.
