# Supplementary material for: Temporal lobe epilepsy is associated with neuroinflammation, extracellular matrix remodeling, and synaptic protein alterations
Source: Front Mol Neurosci. 2026 Feb 20;19:1728666. doi: 10.3389/fnmol.2026.1728666 (PMC12963051; doi:10.3389/fnmol.2026.1728666)
Supplement: Supplementary Figure 1 — Bar chart total amount of identified proteins. [file Data_Sheet_1.docx]

Supplementary Material

# Supplementary Figures and Tables

## Supplementary Figures


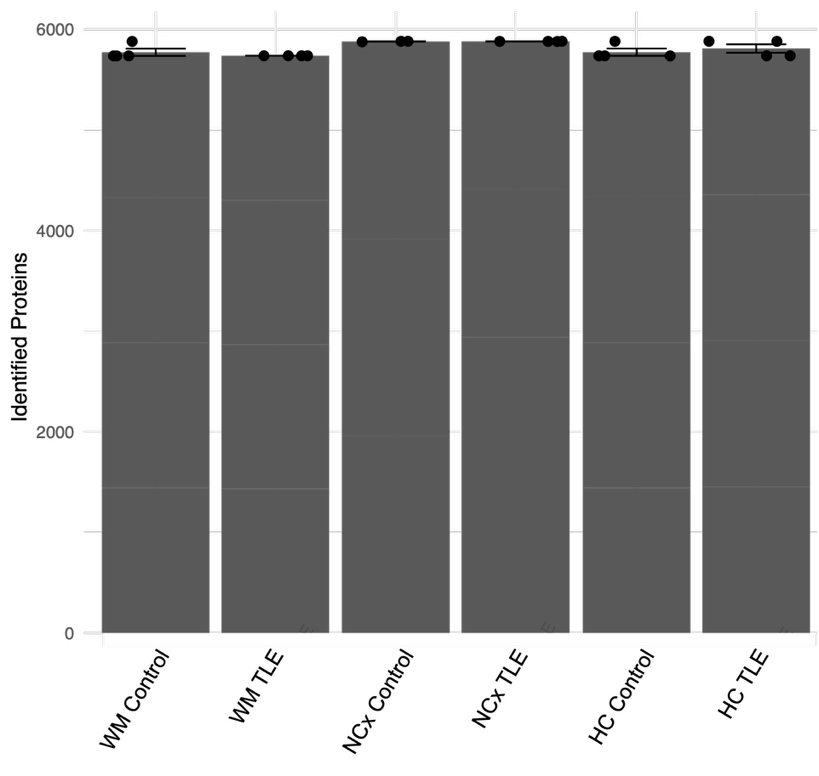


**Supplementary Figure 1.** Bar Chart of the total amount of identified proteins within the sample groups. Each bar corresponds to a distinct sample group, with the y-axis indicating the total number of identified proteins, while the x-axis displays the sample groups, each represented by a dot.


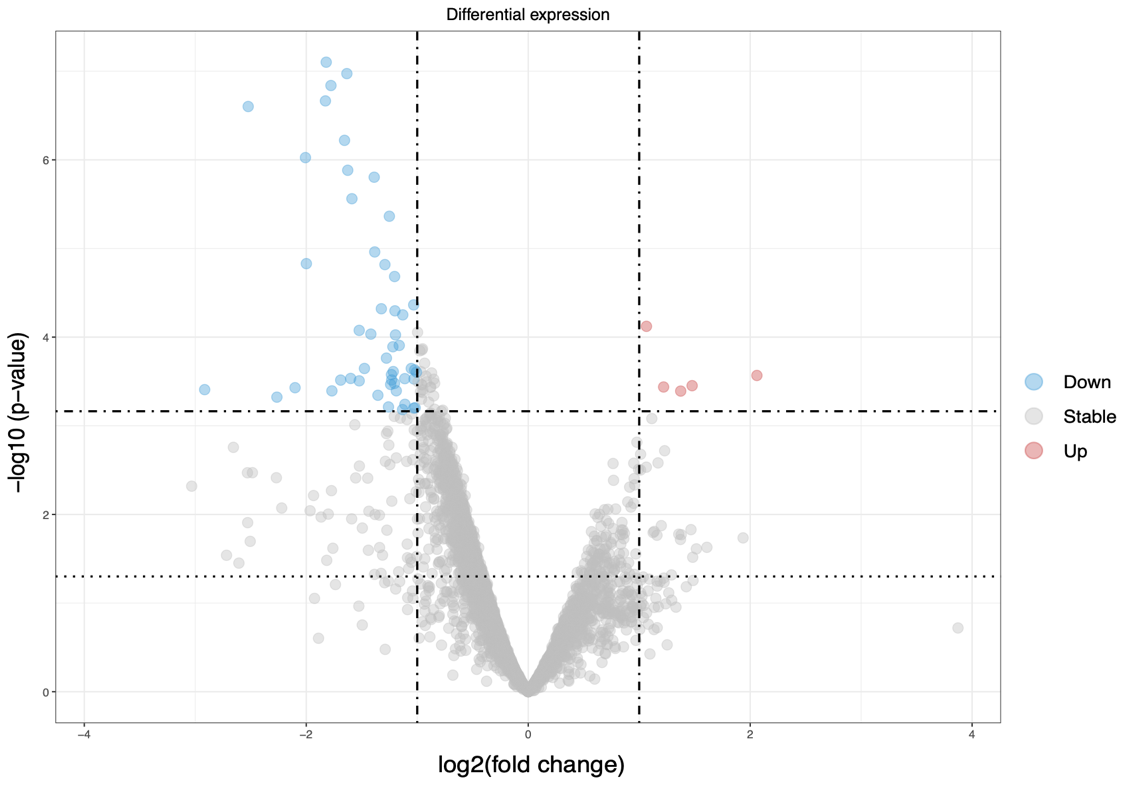


**Supplementary Figure 2. Volcano plot illustrating significantly differentially expressed proteins in white matter tissue in TLE.** Mass-spectrometry based proteomic analysis of white matter tissue of TLE patients and respective control samples were analyzed statistically. The volcano plot illustrates significantly downregulated proteins and significantly upregulated proteins. X-axis shows the log_2_ fold change and y-axis denotates the p-value -log10. Red dots represent significantly upregulated proteins, whereas blue dots mark significantly downregulated proteins. The horizontal dashed lines depict the p-value threshold of 0.05 (lower line) and the adjusted p-value threshold of 0.05 (upper line). The vertical dashed line marks the threshold for an absolute log_2_ fold change ≥1. An interactive version is available upon request.


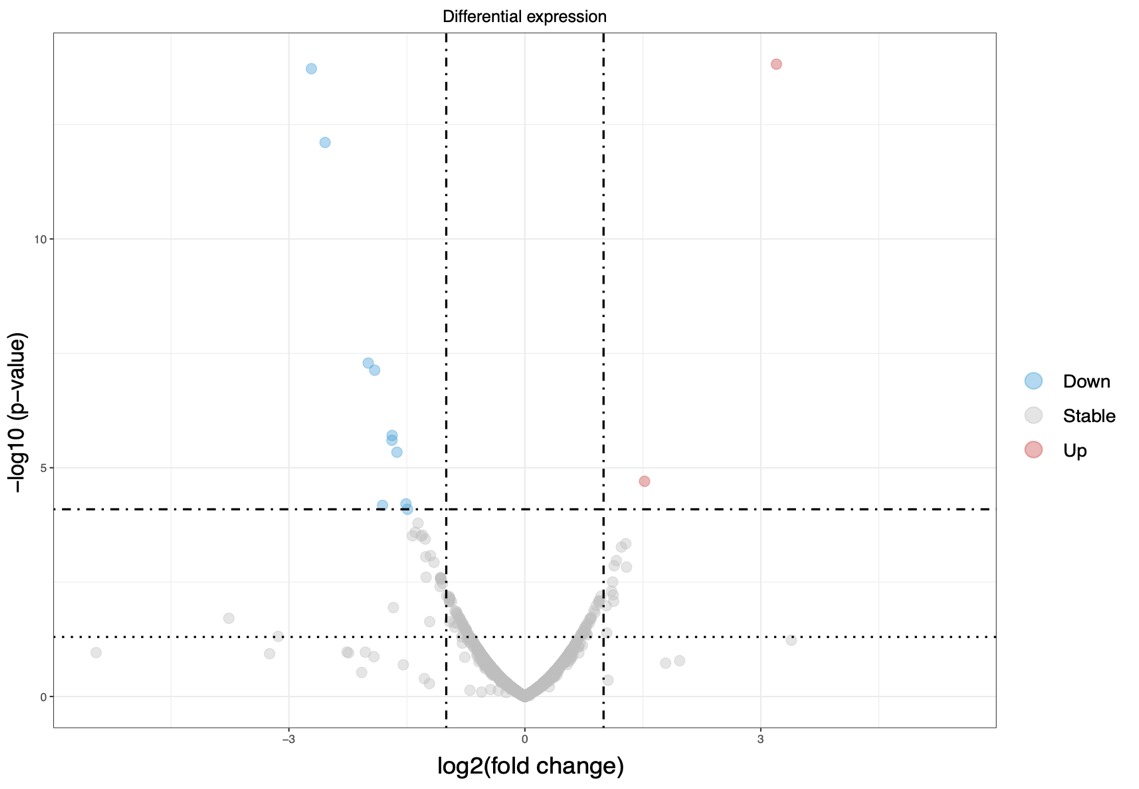


**Supplementary Figure 3. Volcano plot illustrating significantly differentially expressed proteins in neocortical tissue in TLE.** Mass-spectrometry based proteomic analysis of neocortical tissue of TLE patients and respective control samples were analyzed statistically. The volcano plot illustrates significantly downregulated proteins and significantly upregulated proteins. X-axis shows the log_2_ fold change and y-axis denotates the p-value -log10. Red dots represent significantly upregulated proteins, whereas blue dots mark significantly downregulated proteins. The horizontal dashed lines depict the p-value threshold of 0.05 (lower line) and the adjusted p-value threshold of 0.05 (upper line). The vertical dashed line marks the threshold for an absolute log_2_ fold change ≥1. An interactive version is available upon request.

## Supplementary Tables

**Supplementary Table 1.** Statistical Analysis of proteomic differences of the sample comparison groups. The first column lists the respective comparison groups. The second column reports the number of proteins showing nominally significant differential expression (p ≤ 0.05) with an absolute log2 fold change ≥ ±0.5. The third column summarizes proteins remaining significantly differentially expressed after correction for multiple testing using Benjamini-Hochberg procedure (adjusted p ≤ 0.05) and exhibiting an absolute log2 fold change ≥ ±0.9.

| Comparison | p-value ≤ 0.05  \|log_2_ fold change\| ≥ 0.5 | Adj. p-value ≤ 0.05  \|log_2_ fold change\| ≥ 0.9 |
| --- | --- | --- |
| HC Ctrl vs. HC Path TLE | 1193 | 149 |
| WM Ctrl vs. WM Path TLE | 1134 | 80 |
| NCx Ctrl vs. NCx Path TLE | 109 | 13 |

**Supplementary Table 2.** Reactome pathway enrichment analysis of the significantly differentially expressed proteins in the epileptic hippocampus of TLE patients. The table summarizes the most enriched pathways within the significantly upregulated proteins. Column 1 lists the pathway name. Columns 2 and 3 show the nominal p-value and adjusted p-value (following FDR correction for multiple testing), respectively. Column 4 reports the Normalized Enrichment Score (NES), indicating the direction and magnitude of pathway regulation based on the distribution of log2 fold changes of the contributing proteins; a positive NES indicates predominant upregulation. Column 5 (counts) indicates the number of proteins assigned to each pathway.

| **Pathway** | **p-value** | **Adjusted p-value** | **Normalized Enrichment Score** | **Count** |
| --- | --- | --- | --- | --- |
| Complement cascade [R-HSA-166658] | 9,1208E-17 | 6,0562E-14 | 3,72678528 | 33 |
| Regulation of Complement cascade [R-HSA-977606] | 9,1208E-17 | 6,0562E-14 | 3,72678528 | 33 |
| Regulation of IGF Activity by IGFBP [R-HSA-381426] | 6,4194E-15 | 2,1312E-12 | 3,37069358 | 61 |
| Formation of Fibrin Clot (Clotting Cascade) [R-HSA-140877] | 3,7708E-11 | 4,5509E-09 | 3,22531175 | 18 |
| Post-translational protein phosphorylation [R-HSA-8957275] | 3,4561E-12 | 5,0997E-10 | 3,21395059 | 58 |
| Integrin cell surface interactions [R-HSA-216083] | 5,1939E-09 | 3,2845E-07 | 2,94620404 | 37 |
| Common Pathway of Fibrin Clot Formation [R-HSA-140875] | 1,6081E-10 | 1,4237E-08 | 2,93459092 | 10 |
| Metabolism of fat-soluble vitamins [R-HSA-6806667] | 1,404E-07 | 6,9058E-06 | 2,91139663 | 19 |
| Retinoid metabolism and transport [R-HSA-975634] | 1,404E-07 | 6,9058E-06 | 2,91139663 | 19 |
| Class A/1 (Rhodopsin-like receptors) [R-HSA-373076] | 2,71E-07 | 1,1609E-05 | 2,90381046 | 20 |
| Peptide ligand-binding receptors [R-HSA-375276] | 2,71E-07 | 1,1609E-05 | 2,90381046 | 20 |
| Scavenging of heme from plasma [R-HSA-2168880] | 4,553E-09 | 3,0232E-07 | 2,90237074 | 11 |
| Plasma lipoprotein assembly [R-HSA-8963898] | 2,4787E-07 | 1,1351E-05 | 2,84085546 | 13 |
| Binding and Uptake of Ligands by Scavenger Receptors [R-HSA-2173782] | 5,5916E-07 | 2,2502E-05 | 2,80628013 | 25 |
| Initial triggering of complement [R-HSA-166663] | 1,6323E-07 | 7,7417E-06 | 2,79079045 | 15 |
| p130Cas linkage to MAPK signaling for integrins [R-HSA-372708] | 1,6791E-06 | 6,3709E-05 | 2,73919914 | 13 |
| Chylomicron assembly [R-HSA-8963888] | 4,3783E-08 | 2,3258E-06 | 2,7360613 | 9 |
| Visual phototransduction [R-HSA-2187338] | 9,4423E-07 | 3,6881E-05 | 2,7083621 | 32 |
| Intrinsic Pathway of Fibrin Clot Formation [R-HSA-140837] | 2,3163E-06 | 8,5447E-05 | 2,69733085 | 12 |
| Plasma lipoprotein remodeling [R-HSA-8963899] | 2,5746E-08 | 1,4866E-06 | 2,69363464 | 8 |
| GRB2:SOS provides linkage to MAPK signaling for Integrins [R-HSA-354194] | 7,2941E-06 | 0,00023626 | 2,65262977 | 14 |
| Lipoprotein metabolism [R-HSA-174824] | 5,9786E-06 | 0,00019849 | 2,65031477 | 23 |
| Chylomicron remodeling [R-HSA-8963901] | 4,1123E-11 | 4,5509E-09 | 2,64099899 | 7 |
| Platelet Aggregation (Plug Formation) [R-HSA-76009] | 1,5141E-05 | 0,00046762 | 2,57516524 | 23 |
| Regulation of TLR by endogenous ligand [R-HSA-5686938] | 4,2404E-06 | 0,00014439 | 2,55045252 | 8 |
| GPCR ligand binding [R-HSA-500792] | 3,0239E-06 | 0,00010717 | 2,53397607 | 36 |
| Dissolution of Fibrin Clot [R-HSA-75205] | 3,0665E-06 | 0,00010717 | 2,47827415 | 7 |
| Activation of C3 and C5 [R-HSA-174577] | 1,8302E-09 | 1,2792E-07 | 2,47038598 | 6 |
| Syndecan interactions [R-HSA-3000170] | 4,1953E-05 | 0,00121117 | 2,44065661 | 11 |
| Integrin signaling [R-HSA-354192] | 4,4851E-05 | 0,00126727 | 2,40029813 | 22 |
| Hemostasis [R-HSA-109582] | 3,9607E-12 | 5,2598E-10 | 2,38153247 | 197 |
| Response to elevated platelet cytosolic Ca2+ [R-HSA-76005] | 5,6585E-05 | 0,00156551 | 2,36676703 | 9 |
| Extracellular matrix organization [R-HSA-1474244] | 3,4476E-08 | 1,9077E-06 | 2,36231726 | 106 |
| Non-integrin membrane-ECM interactions [R-HSA-3000171] | 0,00011538 | 0,00287385 | 2,33574879 | 24 |
| Platelet degranulation [R-HSA-114608] | 9,4185E-09 | 5,6853E-07 | 2,30791003 | 5 |
| Molecules associated with elastic fibres [R-HSA-2129379] | 0,00010964 | 0,00280009 | 2,3074742 | 9 |
| ECM proteoglycans [R-HSA-3000178] | 0,00040665 | 0,00857184 | 2,25457926 | 30 |
| Diseases associated with the TLR signaling cascade [R-HSA-5602358] | 0,0006723 | 0,01240016 | 2,23903481 | 12 |
| Diseases of Immune System [R-HSA-5260271] | 0,0006723 | 0,01240016 | 2,23903481 | 12 |

**Supplementary Table 3.** Reactome pathway enrichment analysis of the significant differentially expressed proteins in the epileptic hippocampus of TLE patients. The table summarizes the most enriched pathways within the significantly downregulated proteins. Column 1 lists the pathway name. Columns 2 and 3 show the nominal p-value and adjusted p-value (following FDR correction for multiple testing), respectively. Column 4 reports the Normalized Enrichment Score (NES), indicating the direction and magnitude of pathway regulation based on the distribution of log2 fold changes of the contributing proteins; a negative NES indicates predominant downregulation. Column 5 (counts) indicates the number of proteins assigned to each pathway.

| **Pathway** | **p-value** | **Adjusted p-value** | **Normalized Enrichment Score** | **Count** |
| --- | --- | --- | --- | --- |
| Complex I biogenesis [R-HSA-6799198] | 1,7216E-12 | 2,8578E-10 | -2,5607326 | 44 |
| Respiratory electron transport, ATP synthesis by chemiosmotic coupling, and heat production by uncoupling proteins. [R-HSA-163200] | 7,2825E-14 | 1,6119E-11 | -2,4511708 | 86 |
| Respiratory electron transport [R-HSA-611105] | 1,1997E-12 | 2,276E-10 | -2,438109 | 75 |
| The citric acid (TCA) cycle and respiratory electron transport [R-HSA-1428517] | 3,4321E-14 | 9,1157E-12 | -2,4002208 | 108 |
| Mitochondrial translation elongation [R-HSA-5389840] | 4,8629E-11 | 4,8364E-09 | -2,357735 | 64 |
| Mitochondrial translation [R-HSA-5368287] | 5,0986E-11 | 4,8364E-09 | -2,356653 | 68 |
| Mitochondrial translation termination [R-HSA-5419276] | 2,7278E-10 | 2,2641E-08 | -2,3482856 | 63 |
| Mitochondrial translation initiation [R-HSA-5368286] | 8,7984E-10 | 6,4912E-08 | -2,3124259 | 63 |
| Neuronal System [R-HSA-112316] | 4,0465E-16 | 1,7913E-13 | -2,2519904 | 187 |
| Transmission across Chemical Synapses [R-HSA-112315] | 3,5719E-10 | 2,7903E-08 | -2,102552 | 130 |
| Potassium Channels [R-HSA-1296071] | 1,0203E-05 | 0,0003226 | -2,0899853 | 29 |
| Insulin receptor recycling [R-HSA-77387] | 0,00013199 | 0,00312999 | -2,0394725 | 12 |
| Regulation of pyruvate dehydrogenase (PDH) complex [R-HSA-204174] | 0,0002218 | 0,00516746 | -1,9883673 | 10 |
| ROS and RNS production in phagocytes [R-HSA-1222556] | 0,00010241 | 0,00266679 | -1,9872613 | 11 |
| Neurotransmitter receptors and postsynaptic signal transmission [R-HSA-112314] | 4,7412E-07 | 1,9676E-05 | -1,9659808 | 100 |
| GABA receptor activation [R-HSA-977443] | 0,00011686 | 0,00287385 | -1,9507918 | 31 |
| Protein-protein interactions at synapses [R-HSA-6794362] | 1,5779E-05 | 0,00047623 | -1,9365522 | 61 |
| Long-term potentiation [R-HSA-9620244] | 0,00026584 | 0,00598355 | -1,9205856 | 14 |
| RAB GEFs exchange GTP for GDP on RABs [R-HSA-8876198] | 2,179E-05 | 0,00064303 | -1,9192885 | 61 |
